# Aging as a target for the prevention and treatment of Alzheimer’s disease

**DOI:** 10.3389/fneur.2024.1376104

**Published:** 2024-04-05

**Authors:** Lauren E. Yap, James E. Hunt, Raymond Scott Turner

**Affiliations:** Department of Neurology, Memory Disorders Program, Georgetown University, Washington, DC, United States

**Keywords:** aging, mild cognitive impairment, dementia, Alzheimer’s disease, amyloid, caloric restriction, intermittent fasting, resveratrol

## Abstract

Alzheimer’s disease (AD), the most common etiology of dementia in older adults, is projected to double in prevalence over the next few decades. Current treatments for AD manage symptoms or slow progressive decline, but are accompanied by significant inconvenience, risk, and cost. Thus, a better understanding of the risk factors and pathophysiology of AD is needed to develop novel prevention and treatment strategies. Aging is the most important risk factor for AD. Elucidating molecular mechanisms of aging may suggest novel therapeutic targets. While aging is inevitable, it may be accelerated by caloric excess and slowed by caloric restriction (CR) or intermittent fasting. As such, CR may slow aging and reduce the risk of all diseases of aging, including dementia due to AD. The literature on CR, intermittent fasting, and treatment with polyphenols such as resveratrol—a pharmacologic CR-mimetic—supports this hypothesis based on clinical outcomes as well as biomarkers of aging and AD. More studies exploring the role of CR in regulating aging and AD progression in man are needed to fill gaps in our understanding and develop safer and more effective strategies for the prevention and treatment of AD.

## Introduction

Alzheimer’s disease (AD), a gradually progressive neurodegenerative disorder, is the most common cause of dementia in the elderly. Dementia due to AD currently affects 6.7 million Americans over the age of 65, and the prevalence of AD is projected to double to 12.7 million by 2050 (1). As the number of older adults with AD increases, so does the need for safe and effective strategies for its prevention and treatment. Traditional treatments (administered by mouth or by transdermal patch) support CNS cholinergic neurotransmission (cholinesterase inhibitors) or block excitotoxic neuronal injury (memantine) and provide temporary, symptomatic, and palliative benefits. Recently-approved anti-amyloid beta (Aβ) monoclonal antibodies clear CNS amyloid and slow the rate of decline but are accompanied by inconvenience (parenteral administration), risk, and cost. Novel prevention and treatment strategies for AD are sorely needed.

While aging is the most important risk factor for AD, age-related molecular mechanisms leading to AD pathologies in brain remain unclear. Thus, a better understanding of the pathophysiology of AD through preclinical and human research is needed to identify novel molecular targets and regulatory pathways. Here, we review the modulation of aging by caloric restriction (CR) or intermittent fasting in man as a potential strategy to prevent or slow the progression of AD and propose studies designed to fill gaps in our current knowledge.

## Pathophysiology and classification of Alzheimer’s disease

AD is classified on a continuum from preclinical AD (normal cognition) to mild cognitive impairment (MCI) and lastly, dementia (mild, moderate, and severe). Patients in the preclinical stage have no cognitive signs or symptoms but positive AD biomarkers, while patients with MCI exhibit preserved function but cognitive decline beyond normal aging. Dementia is defined by both cognitive and functional impairments. The pathology of AD includes the progressive accumulation and deposition of abnormal proteins in the brain, including Aβ amyloid (plaques) and phosphorylated tau (tangles), as well as inflammatory responses (gliosis) and the loss of synapses, neurotransmitters, and neurons (atrophy). Clinically, AD manifests with progressive amnesia (especially episodic memory), word-finding pauses, and difficulty performing complex activities of daily living, while later stages include more severe cognitive deficits impairing all basic activities of daily living.

AD was traditionally diagnosed based on clinical signs and symptoms and/or pathology on postmortem brain examination. Recently, however, studies reveal a lack of correlation between clinical expression and early neuropathologic changes (particularly amyloid burden) in individuals with AD (2). This prompted a shift in the definition of AD to include a longer clinical-biological continuum leading to dementia. Beyond the traditional clinicopathological features, AD may be further categorized by the ATX(N) system, which includes AD biomarkers that are readily detectable in living individuals: (1) Plaques composed of Aβ/amyloid (A), (2) neurofibrillary tangles composed of phosphorylated tau (T), (3) novel biomarkers including immune dysregulation and synaptic dysfunction (X), and (4) neurodegeneration (N) (2–4). These biomarkers may be employed in screening, diagnosis, prognosis, disease staging, efficacy assessment, and target engagement of novel treatments.

## Risk factors for Alzheimer’s disease

The major risk factors for AD are: (1) aging, (2) genetics and family history of dementia or AD, and (3) diabetes mellitus/obesity/metabolic syndrome (especially during midlife). Aging and family history/genetics are considered non-modifiable risk factors. We hypothesize, however, that *aging* (and AD) may be slowed by modulation of caloric intake/glucose metabolism via *epigenetic* regulation—ultimately altering Aβ metabolism and its downstream consequences. Thus, aging and genetics may, in fact, be modifiable risk factors.

Aging, defined as a change in a biological parameter as a function of time, is clearly the most important risk factor for AD, perhaps due to slowing of CNS Aβ clearance (particularly in the sporadic form of AD). Genetics and family history of dementia/AD constitute the second most important risk factor. The *APOE4* variant of *ApoE* is the strongest genetic risk factor for sporadic AD—the form that accounts for >95% of cases (5). While the role of this gene and protein in the brain is not completely understood, *APOE* variants are associated with changes in gene expression, particularly involving cholesterol homeostasis and transport signaling pathways. This results in aberrant deposits of cholesterol in oligodendrocytes and decreased myelination - found in postmortem human brain (5). With regards to early-onset familial AD, mutations in the *amyloid precursor protein (APP)* gene are associated with 10–15% of familial cases (6). In human astrocytes, APP is involved in the endocytosis of low-density lipoprotein receptor ligands in addition to cholesterol homeostasis (7). Mutations in *APP* can lead to impaired cholesterol metabolism, perhaps contributing to AD pathogenesis.

Diabetes mellitus and obesity/metabolic syndrome, particularly during midlife, are the third most important risk factors for AD. In animal models, persistent hyperglycemia leads to chronic inflammation and neuroinflammation (8). Patients with Type 2 diabetes mellitus who take anti-diabetic agents, such as metformin and glucagon-like peptide 1, have a reduced risk for concomitant AD as well as all-cause dementia (8). Additionally, diabetes and obesity are associated with pro-inflammatory states through the effects of a high-fat diet. In mouse models, this diet triggers insulin resistance in addition to the accumulation of Aβ and hyperphosphorylated tau aggregates in the brain (9).

## Theories and mechanisms of aging

Aging occurs in all living organisms and is typically characterized by a decline in a cellular or tissue function over time (4). While aging *per se* is not well understood, animal and human studies describe several intertwined molecular, cellular, and systemic mechanisms of aging that may be involved in the pathogenesis of age-related diseases, including AD. At the molecular level, genomic instability, epigenetic alterations, and oxidative injury are all implicated in aging (4, 10). Genomic instability due to DNA mutations and disruptions in DNA repair may also contribute to the development of AD. Specifically, DNA damage overwhelms repair capacity, leading to the mistranslation of DNA (4, 11). As DNA damage accumulates in neurons, increased oxidative stress and inflammation promote neurodegeneration, senescence, and AD. In addition, aging influences epigenetic modifications, such as DNA methylation, which results in DNA damage and neurodegeneration, both of which contribute to AD progression (4).

Functional autophagy is crucial in removing damaged mitochondria, regulating intracellular proteins, and clearing misfolded proteins including Aβ/amyloid and tau/tangles (4, 12). However, both aging and AD compromise this key lysosome-based proteolytic pathway, leading to a deleterious cycle of heightened oxidative damage, Aβ and tau accumulation, synaptic dysfunction, and cognitive impairment (4, 13). Evidence in support of these mechanisms includes a human study demonstrating slowing of Aβ turnover as a function of age; over five decades, the half-life of cerebrospinal fluid (CSF) Aβ increases ~2.5-fold (14). Thus, while autosomal dominant mutations (in *APP*, *PS1*, or *PS2*) causing familial AD result to Aβ overproduction, aging associated with sporadic AD leads to impaired Aβ clearance. The resulting imbalance between production and clearance results in the progressive accumulation and deposition of Aβ in the brain parenchyma (amyloid plaques) and blood vessel walls (amyloid angiopathy).

Cellular and systemic mechanisms may also underlie the role of aging in the development and progression of AD. At the cellular level, senescence, stem cell exhaustion, and altered intercellular communication may contribute to aging (4, 10). Aβ oligomerization also influences key pathways involved in aging; these neurotoxic extracellular aggregates interfere with signal transduction and synaptic plasticity (15). Lastly, at a systemic level, aging may be propelled by deregulated sensing of nutrients and a chronic inflammatory state (4, 10).

## Regulation of aging

**Figure 1:**
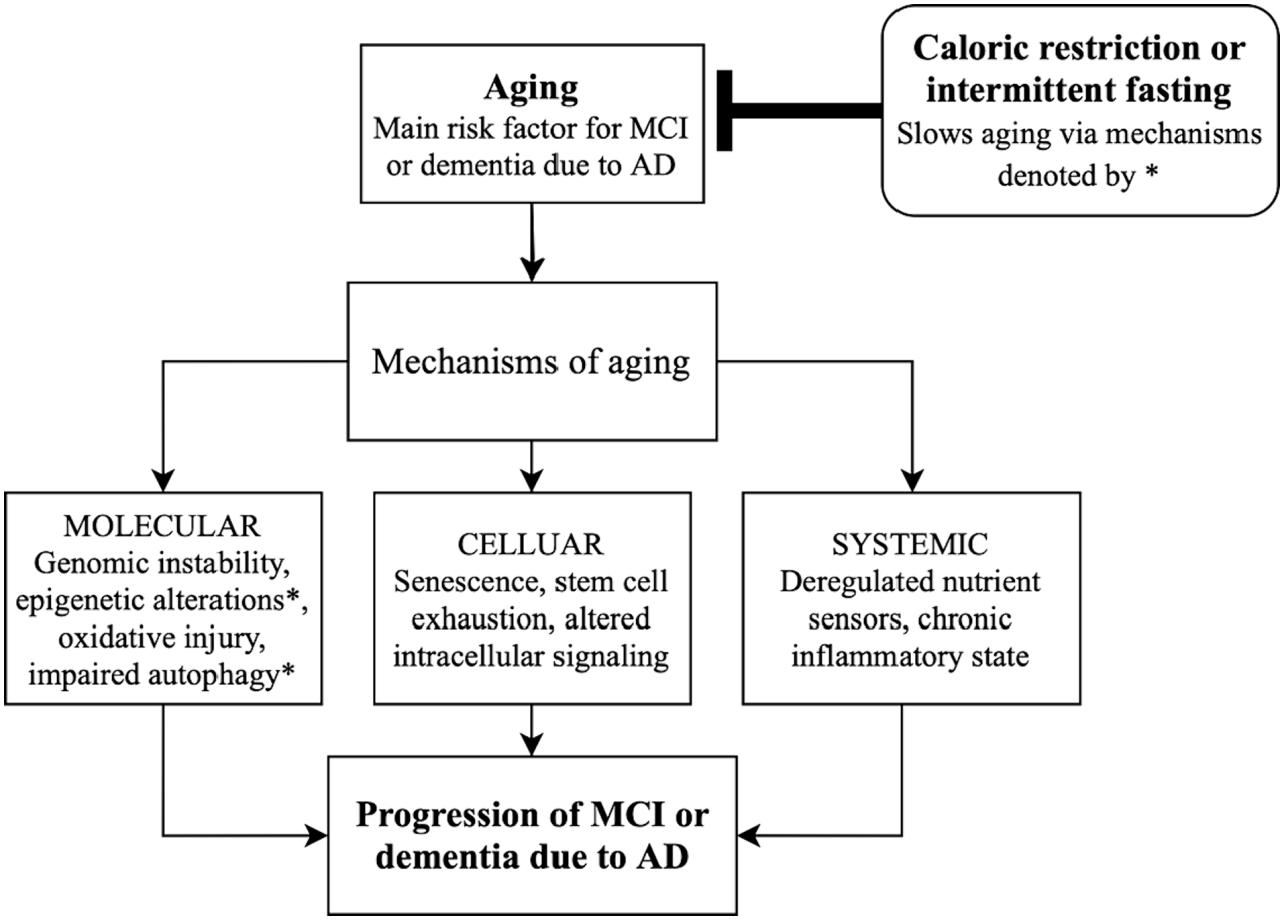
Schematic diagram linking caloric restriction and intermittent fasting with slowing the progression of Alzheimer’s disease by altering mechanisms of aging. AD, Alzheimer’s disease; MCI, mild cognitive impairment.

While aging may be inevitable, certain factors regulate its course. Aging may be accelerated by caloric excess—a notion supported by a human study demonstrating an association between obesity and the expression of 21 genes related to AD (16). If states of caloric excess, including diabetes, obesity, and metabolic syndrome accelerate aging, then its inverse—caloric restriction (CR)—may slow aging and prevent or delay diseases of aging. While there are few human studies exploring the effect of CR on AD, preclinical studies with animal models support this hypothesis. In mice, long-term CR has a neuroprotective effect by inducing autophagy, leading to the degradation of Aβ and other aggregated proteins [Figure 1, (17)]. Moreover, markers of autophagic activity are simultaneously increased as lipid peroxidation and apoptosis are decreased in AD-related brain regions when mice receive a CR regime via prolonged intermittent fasting (12).

Along with controlling autophagy, caloric intake may influence epigenetic regulation (Figure 1). Sirtuins (SIRTs) are genes/enzymes that link energy balance—regulated by the intracellular NAD+/NADH ratio—to epigenetic regulation of gene expression. Such mechanisms include histone deacetylation, which impacts gene expression and may be essential in the effect of CR on slowing aging (18, 19). Mammalian SIRT1 deacetylates transcription factors which are responsible for controlling metabolic pathways, making pharmacologic targets of SIRT1 of particular interest for regulating energy metabolism and aging (18, 19).

## Role of caloric restriction and time-restricted eating in Alzheimer’s disease

We hypothesize that CR (and its pharmacologic mimics) may be safe and effective in both the prevention and treatment of individuals within the AD spectrum. This notion is supported by the following human studies.

### Caloric restriction

A post-hoc analysis of the Comprehensive Assessment of Long-term Effects of Reducing Intake of Energy (CALERIE) trial explored the effects of reducing caloric intake by 25% in healthy, non-obese adults for two years (19). The investigators explored changes in epigenetic modifications associated with aging. The analysis utilized established algorithms to estimate both the biological age and rate of aging based on DNA methylation (DNAm) in blood samples. PhenoAge and GrimAge are DNAm clocks that estimate biological age based on a reference population. The investigators demonstrated that PhenoAge and GrimAge values were not significantly different between calorie reduction and control groups. While PhenoAge and GrimAge estimate biological age, DunedinPACE is an algorithm that estimates the rate of aging, which is defined as the years of biological aging undergone during one calendar year. The analysis demonstrated that a 25% calorie reduction significantly decreased the pace of aging (Table 1) (20). By slowing the rate of aging via epigenetic modifications, CR may lower the risk of age-related diseases including AD.

**Table 1:** Summary of human studies on the effect of caloric restriction or its mimetics on Alzheimer’s disease outcomes.

Study	Intervention	Study type and participants	AD biomarker outcome	Mechanisms of aging outcome	Cognitive outcomes	Conclusion
Waziry et al. (20)	25% calorie reduction for 2 years	Post-hoc analysis of the Comprehensive Assessment of Long-term Effects of Reducing Intake of Energy (CALERIE) trial, phase II, multicenter, randomized controlled trial; n = 220 healthy adults (men 21–50 years old, women 21–47 years old)	—	Based on serum DNA methylation levels, significantly decreased the pace of aging (DunedinPACE algorithm; *p* = <0.003) but no significant difference in biological age estimation (PhenoAge and GrimAge)	—	25% calorie reduction decreased the rate of aging, however, did not lead to changes in biological age estimation
Horie et al. (21)	Nutritional counseling on weight loss via CR in group meetings for 12 months	Single-center prospective randomized controlled trial; n = 80 older adults (>60 years old) with obesity and MCI	—	Reduced HOMA-IR and CRP were associated with improved global cognition and an increase in delayed memory, respectively; increased leptin correlated with improved attention	Decreased BMI was associated with improved verbal memory, fluency, executive function, and global cognition via RAVLT and TMT	Intentional weight loss through CR correlated with improved cognition in older adults with MCI, and the strongest association was with younger seniors and APOE e4 carriers
Turner et al. (18), Sawda et al. (19)	Resveratrol (final dose of 1,000 mg BID) - caloric restriction mimic via SIRT1 activation	52-week randomized, double-blind, placebo-controlled phase II trial; n = 104 adults >49 years old with mild-to-moderate AD	Significantly higher Aβ40 levels in CSF and plasma in the resveratrol-treated group (*p* = 0.002)	—	Reduced decline in ADCS-ADL scores in the resveratrol-treated group, although not significant (underpowered)	Resveratrol stabilizes plasma and CSF Aβ40 levels
Moussa et al. (22)	Resveratrol (final dose of 1,000 mg BID)	Retrospective subgroup study of Turner et al. (18); analyzed CSF and plasma samples from a subset of participants with CSF biomarker-proven AD at baseline (CSF Aβ42 < 600 ng/mL); n = 19 resveratrol-treated, n = 19 placebo-treated	Greater decline of CSF Aβ42 in the placebo group than resveratrol group (*p* = 0.0618)	In CSF, ~50% decline in CSF MMP-9 and increased MDC, IL-4, FGF-2 in resveratrol group after 52 weeks; in plasma, increased levels of MMP-10 and decreased IL-12P40, IL12P70, and RANTES with resveratrol treatment	Reduced decline in MMSE and ADCS-ADL scores in the resveratrol group	Resveratrol slows the progression of AD via its effects on regulating neuroinflammation and inducing adaptive immunity
Ooi et al. (23)	Intermittent fasting (IF) practiced regularly or irregularly (control: not fasting)	Longitudinal study of n = 99 older adults (>60 years old) with mild cognitive impairment (MCI) and otherwise healthy; maximum follow-up 36 months	—	Increased superoxide dismutase activity, decreased body weight, insulin levels, CRP, and DNA damage with regular IF	Increase in mean scores for Digit Span Test, RAVLT, MMSE, and MOCA in fasting groups	Regularly practiced IF led to improved cognitive scores and cognitive function in older adults with MCI

Horie et al. (21) conducted a single-center randomized controlled trial to investigate the effect of nutritional counseling (centered on weight loss through CR) on cognitive outcomes. Neuropsychological testing included the Rey Auditory Verbal Learning Test (RAVLT) for verbal memory and the Trail Making Test (TMT) for attention, working memory, and psychomotor processing speed. Adults (60+) with obesity and MCI exhibited improvement in cognitive tests after one year (Table 1). On average, the body mass index (BMI) decreased by 1.7 ± 1.8 kg/m^2^ (*p* = 0.02), and a decrease in BMI correlated with improved verbal memory and fluency, executive function, and global cognition. Moreover, there were associations between aging biomarkers and cognition: decreased homeostasis model assessment-estimated insulin resistance (HOMA-IR) was associated with improved global cognition and fluency; reduced C-reactive peptide (CRP) levels correlated with increased delayed memory; and increased leptin, a hormone that suppresses appetite, correlated with improved attention (21). Overall, obese participants with MCI who intentionally lost weight through CR demonstrated improved cognition, potentially via molecular mechanisms of aging.

### Resveratrol

A phase II multicenter, randomized study investigated the effects of resveratrol, a SIRT1 regulator, on clinical outcomes and biomarkers of AD as well as mechanisms of aging (18, 19). Resveratrol is a polyphenol found in red grapes, red wine, and other foods. As an activator of SIRT1, resveratrol acts as a pharmacologic CR-mimetic. Resveratrol dosage was titrated to a final dose of 1,000 mg (oral) twice daily over 52 weeks. We found a difference in Aβ40 levels in CSF and plasma between the resveratrol-treated group and placebo group (*p* = 0.002; Table 1). While the placebo group experienced a decline in Aβ40 levels during the study (which correlates with disease progression) the resveratrol-treated group had stable Aβ40 levels in CSF and plasma. Similar trends were found with Aβ42 levels in CSF and plasma (18, 19).

In addition to SIRT activation, resveratrol may play a role in regulating other processes of aging and AD, including decreased inflammation, oxidative stress, and Aβ aggregation (19). CSF matrix metalloproteinase 9 levels (MMP-9) declined by approximately 50% in the resveratrol-treated group during the trial. MMP-9 regulates the permeability of the blood–brain barrier by cleaving tight junctions and releasing cytokines and free radicals. Decreased CSF MMP-9 levels suggest that resveratrol may preserve blood–brain barrier integrity and decrease its permeability to proinflammatory mediators. From a safety perspective, resveratrol is well-tolerated. The only side effects noted were weight loss, brain pseudoatrophy, and a lower incidence of cancer (19). Taken together, resveratrol (or similar compounds targeting sirtuins) may be a promising option in stabilizing the progression of AD and decreasing CNS inflammation by promoting blood–brain barrier integrity.

A subset analysis was subsequently conducted using banked CSF and plasma samples collected from CSF biomarker-confirmed AD participants only (22). The investigators compared markers of neurodegeneration, MMPs, and cognitive outcomes in AD participants who were treated with resveratrol versus placebo. After 52 weeks, treated participants exhibited decreased CSF levels of MMP-9 (as mentioned above), as well as macrophage-derived chemokine (MDC), interleukin (IL)-4, and fibroblast growth factor (FGF)-2. Additionally, plasma levels of MMP-10 were increased and IL-12P40, IL12P70, and RANTES were decreased in the resveratrol-treated group. Taken together, these results indicate that resveratrol plays a role in regulating neuroinflammation and promoting adaptive immunity. Treatment with resveratrol also yielded favorable cognitive outcomes in this subset analysis: while MMSE scores declined in the placebo group after 52 weeks (*p* < 0.01), there was no change in MMSE scores in the resveratrol-treated group. Likewise, the Alzheimer’s Disease Cooperative Study-Activities of Daily Living (ADCS-ADL) scores of the placebo group decreased by two orders of magnitude more than those of the resveratrol treated group, suggesting that resveratrol may attenuate decline in both cognition and function.

### Intermittent fasting

Due of the difficulty in adhering to a strict CR protocol in humans, some studies utilize intermittent fasting (IF) as a more feasible alternative (24). In a longitudinal study of adults (60+) with MCI, regular IF resulted in improved mean scores on the Digit Span Test, RAVLT, Mini-Mental State Examination (MMSE), and Montreal Cognitive Assessment (MoCA) (Table 1) (23). While specific AD biomarkers were not assessed, IF was associated with changes in oxidative stress, inflammation, and DNA integrity, which are all implicated in aging. Specifically, participants who regularly practiced IF exhibited increased superoxide dismutase activity and decreased body weight, insulin levels, CRP, and DNA damage (23). In summary, regular IF in older adults with MCI improved cognitive function as well as altered markers of aging.

## Ongoing and future clinical studies

Studies in progress relevant to this brief review include: CR of obese patients with MCI (clinicaltrials.gov NCT# 01286389), and CR, resveratrol, and dietary interventions on the aging brain (00996229, 01219244). Pharmacologic trials in progress enrolling individuals in the AD spectrum include: metformin (04511416, 04098666), metformin with lifestyle/dietary modifications (05109169), intranasal insulin/empagliflozin (05081219), liraglutide (05313529), semaglutide (04777396, 04777409; isrctn.com # ISRCTN71283871), dasatanib/quercetin (04685590, 05422885), canakinumab (04795466), and nicotinamide (05617508, 04430517, 05040321). Additional strategies under investigation include bariatric surgery, mTOR inhibitors, epigenetic therapies, telomerase modulators, probiotics, and mesenchymal stem cells.

Short-term CR, such as a 7-day inpatient stay in a clinical research unit, is feasible with human subjects. To investigate CR as a strategy to slow aging and AD pathogenesis, admitted subjects would have caloric intake strictly regulated and monitored. Since Aβ levels in CSF and plasma are dynamic, with a short half-life (a few hours), we hypothesize that short-term CR may alter CSF and plasma Aβ metabolism. Clinical studies are also warranted with the newer treatments for diabetes and weight loss (DDP-4 inhibitors, GLP-1 receptor agonists, and SGLT2 inhibitors). In these proposed clinical trials, aging biomarkers, AD biomarkers in the ATX(N) schema, and clinical outcomes would be collected and analyzed.

## Conclusion

While a mayfly has a lifespan of one day, an elephant’s lifespan may exceed 100 years. Clearly, lifespan and aging are biological traits regulated by genetics and molecular signaling pathways – that may be exploited as a therapeutic target. Aging, however, is not recognized as a disease by the U.S. Food and Drug Administration. Thus, there are no FDA-approved treatments specifically for aging. Aging, however, is the most important risk factor for multiple diseases, including dementia and AD. As molecular mechanisms regulating aging are coming to light and signaling pathways uncovered, novel therapeutic targets present an alternative approach: instead of targeting one disease at a time—leading to the inconvenience, cost, and risk of polypharmacy—targeting aging directly may prevent or slow multiple age-related diseases. CR may be a promising preventive or therapeutic option for individuals at risk for AD or already within the AD spectrum. However, current data are limited and human studies are scarce (hence this mini-review). Additional preclinical and human studies are now warranted to discover the pathways regulated by CR and to identify pharmacophores that mimic the beneficial effects of CR.

Hypotheses linking CR and weight loss to alterations in biomarkers of aging and AD may suggest novel treatment targets and strategies—and not just for AD. Newly discovered therapies may be safe and effective for prevention and/or as an adjunct to FDA-approved treatments for individuals in the AD spectrum. Prevention and treatment strategies targeting aging may be safer and more effective than the currently available treatments targeting more downstream pathways. While several studies are in progress (listed above), more are needed. In the meantime, AD trials should consider including biomarkers of aging and aging studies should include AD biomarkers.

## Author contributions

LY: Conceptualization, Data curation, Investigation, Methodology, Supervision, Writing – original draft, Writing – review & editing. JH: Data curation, Investigation, Writing – original draft, Writing – review & editing. RT: Conceptualization, Data curation, Investigation, Methodology, Supervision, Writing – original draft, Writing – review & editing.
